# Evaluation of a system of structured, pro-active care for chronic depression in primary care: a randomised controlled trial

**DOI:** 10.1186/1471-244X-10-61

**Published:** 2010-08-04

**Authors:** Marta Buszewicz, Mark Griffin, Elaine M McMahon, Jennifer Beecham, Michael King

**Affiliations:** 1 Research Department of Primary Care & Population Health, University College London (Archway Campus), Highgate Hill, London N19 5LW, UK; 2 Academic Department of Psychiatry, University College London (Royal Free Campus), Rowland Hill Street, London NW3 2PF, UK; 3 Personal Social Services Research Unit, London School of Economics and Political Science, Houghton Street, London WC2A 2AE, UK & University of Kent, Cornwallis Building, Canterbury, Kent CT2 7NF, UK

## Abstract

**Background:**

People with chronic depression are frequently lost from effective care, with resulting psychological, physical and social morbidity and considerable social and financial societal costs. This randomised controlled trial will evaluate whether regular structured practice nurse reviews lead to better mental health and social outcomes for these patients and will assess the cost-effectiveness of the structured reviews compared to usual care.

The hypothesis is that structured, pro-active care of patients with chronic depression in primary care will lead to a cost-effective improvement in medical and social outcomes when compared with usual general practitioner (GP) care.

**Methods/Design:**

Participants were recruited from 42 general practices throughout the United Kingdom. Eligible participants had to have a history of chronic major depression, recurrent major depression or chronic dsythymia confirmed using the Composite International Diagnostic Interview (CIDI). They also needed to score 14 or above on the Beck Depression Inventory (BDI-II) at recruitment.

Once consented, participants were randomised to treatment as usual from their general practice (controls) or the practice nurse led intervention. The intervention includes a specially prepared education booklet and a comprehensive baseline assessment of participants' mood and any associated physical and psycho-social factors, followed by regular 3 monthly reviews by the nurse over the 2 year study period. At these appointments intervention participants' mood will be reviewed, together with their current pharmacological and psychological treatments and any relevant social factors, with the nurse suggesting possible amendments according to evidence based guidelines. This is a chronic disease management model, similar to that used for other long-term conditions in primary care.

The primary outcome is the BDI-II, measured at baseline and 6 monthly by self-complete postal questionnaire. Secondary outcomes collected by self-complete questionnaire at baseline and 2 years include social functioning, quality of life and data for the economic analyses. Health service data will be collected from GP notes for the 24 months before recruitment and the 24 months of the study.

**Discussion:**

558 participants were recruited, 282 to the intervention and 276 to the control arm. The majority were recruited via practice database searches using relevant READ codes.

**Trial registration:**

ISRCTN36610074

## Background

Major depression is very common, with a UK prevalence at any time of at least 5%, with another 5% having milder episodes [1]. The majority of people with depression in the UK are treated within general practice, it being the third most common reason for consultations [2]. Despite evidence that over half of all patients with an acute depressive episode will have a recurrence, and that the risk of further recurrences increases greatly with further episodes, there appears to be little consistency in the longer-term management of the disorder, and significant psychological, physical and social morbidity in this group [3,4]. In addition, a significant minority of patients (around 18-25%) will have chronic depressive disorders [3]. Chronicity is associated with greater likelihood of psychological, physical and social morbidity, an earlier death from all causes, and the health and social costs are considerable [5,6]. Incomplete recovery increases the risk of relapse and may predict long-term outcome more accurately than baseline severity [7,8]. Evidence also shows that the earlier a recurrence is detected, the better and speedier the recovery, but currently many such patients may be inadequately treated and have little or no specific follow-up in primary care [9,4].

The rationale behind this trial was the treatment of depression as a potentially chronic or recurring problem, using regular pro-active contact and follow-up of at risk patients by practice nurses, supported by general practitioners (GPs) in their practices [10]. There is evidence in favour of such strategies from the USA, but they have not yet been formally researched in the UK [11]. Work from the USA has shown that organised, enhanced care can have a beneficial effect both on the outcomes of patients with acute major depression and also those with a high risk of recurrence [12,13]. However, there is also some evidence that the effect of such a coordinated approach can lapse over time and recent work has indicated that a longer-term approach may be indicated, particularly for those at risk of chronic difficulties [14,15].

The form of organised or enhanced care being trialled has elements in common with the management of other chronic diseases in general practice, such as asthma, diabetes and hypertension [10]. Evidence based guidelines identify similar elements of patient care for a range of chronic conditions, including a well-defined care plan, patient education, scheduled follow-ups, review of outcome and concordance, and targeted use of specialist consultation or referral [16]. Practice nurses are in an excellent position to provide such an approach and there is evidence that they can do this very well, often communicating particularly effectively with patients in the management of chronic problems [17].

## Feasibility Work

A 6-month pilot feasibility study was conducted in three general practices in North London, with 35 patients randomised by practice. The aim was to test out the intervention and the measures to be used in a full trial and to conduct qualitative, in-depth interviews with 12 patients who completed the intervention. Participants responded well to this practice nurse intervention, particularly valuing contact with someone who they perceived as a 'normal, mature person drawing on their own experience', rather than with a mental health professional. Practice nurses had more time than GPs, gave helpful, practical advice and a sense that they were interested in patients' problems [18].

This pilot study demonstrated that it was feasible to recruit sufficient eligible participants willing to take part and that they would be able to complete the relevant questionnaires. The feasibility study also indicated that the chances of contamination between intervention and control patients within a practice was low, as the majority of the patient contact in the intervention was carried out by nurses who were very unlikely have consultations with the control patients to discuss their mental health symptoms, although they might carry out other practice nurse consultations for physical health reasons. We therefore decided to randomise by patient within practices in the main trial, rather than carrying out a cluster randomisation design.

## Methods/Design

This is a randomised controlled trial, with randomisation by patient within practices. The comparison is between 'GP usual care' (control arm), and a 'structured care' approach involving regular follow-up by practice nurses (intervention arm) in addition to GP usual care, for patients with a history of recurrent or chronic depression.

**Figure 1:**
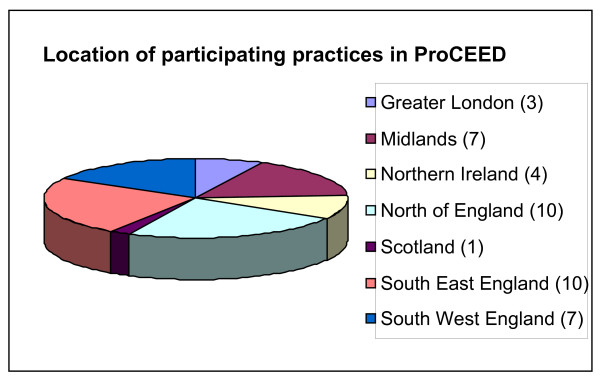
**Location of participating practices**.

Participants were recruited from 38 general practices which are members of the Medical Research Council's General Practice Research Framework (MRC GPRF), a framework of over 1,000 general practices throughout the United Kingdom. An additional 4 practices were recruited, having received details of the study and expressing an interest in participating (2 from the UK's Primary Care Research Network and 2 from local contacts). See Figure 1 for the distribution of practices nationally.

Within practices potential trial participants were recruited in the following ways:

• General practitioner and practice nurse recall of potential participants

• Practice database searches by READ code (a clinical classification system widely used in UK primary care) and/or anti-depressant prescriptions [19].

• Practice leaflets and posters in the waiting-room encouraging patients to come forward

For the full inclusion and exclusion criteria see below. This was a pragmatic trial, so we aimed to keep the exclusion criteria to a minimum. Patients who expressed suicidal ideas were not excluded, but the practice nurses were given clear guidelines about their management and at what point it would be appropriate to communicate with the GP if they were concerned about a patient.

### Inclusion criteria

(i) Men and women aged 18 and over

(ii) Two or more documented episodes of depression within the previous 3 years

(These may have been separate episodes with a period of well-being in-between, or a documented history of chronic depression. In either case some treatment, either pharmacological or psychological or both should have been instigated, although it may not have been successful)

(iii) Evidence of recurrent and/or chronic depression via the Composite International Diagnostic Interview [20]

(iv) Baseline Beck Depression Inventory-II score of 14 or above (indicating at least mild depression) [21].

(We selected this inclusion criterion to cover patients with chronic dysthymia and those with residual symptoms from major depression, as there is much evidence that this group is at particular risk of recurrence and poor outcomes)

(v) Sufficient English to be able to complete self-report questionnaires

### Exclusion criteria

(i) Current psychotic symptoms

(ii) Impaired cognitive function

(iii) Incapacitating alcohol or drug dependence

All identified patients were sent a letter from the GP and practice nurse on practice headed notepaper, enclosing a study information leaflet and a reply slip and asking them to indicate whether they would like to meet with the practice research nurse to discuss the study in more detail. Those who declined were asked to give an indication of their reasons for this on the reply slip.

Those who met with the nurse and still wanted to take part after discussing the study were assessed for eligibility using the Composite International Diagnostic Interview (CIDI) questionnaire and given a Beck Depression Inventory (BDI-II) questionnaire to complete [20,21]. Those who fulfilled the inclusion criteria and gave fully informed consent had their details passed to an independent computerised telephone/internet based randomisation centre. The nurse was able to let participants know their randomisation outcome at the baseline interview. All eligible consented participants in both groups were asked to complete the baseline questionnaires in a quiet place in the practice centre before they left, with the aim of achieving a high response rate.

### Randomisation

We used the MRC computerised telephone randomisation service in Aberdeen, which provides an efficient automated 24-hour service and the choice of randomisation by telephone or Internet in each case. Randomisation was at the patient level within each practice, using a block design to maintain a balance of numbers in the control and intervention groups.

### Trial Intervention

The trial intervention aims to combine management strategies already familiar to primary care staff, but in a concerted and consistent manner [22]. It was found to be well within the expertise of the practice nurses involved in the feasibility study. The model is similar in some ways to that used in the longer-term care of patients with asthma or diabetes in primary care. Components include:

### Recall system

Recall systems have been set up within the participating practices based on the practice computer and managed by the practice research nurse, who is responsible for contacting the intervention patients. If participants fail to attend a review appointment they are asked to make another appointment. If they fail to attend again, they are contacted for review at the next time point.

### Clinical review

For all intervention participants the practice research nurse undertook a baseline assessment, asking about current mood, social circumstances, current treatment (medication and/or psychological therapy), and any side-effects or queries. Participants were given a specially written educational booklet about depression and its treatment at this initial appointment. There is evidence that appropriate patient education materials can be helpful as part of an integrated approach' [11-13]. This patient booklet was written specifically for the patients in this study and outlines current evidence based thinking about the treatment of depression.

The nurses answered participants' questions about current or past treatments and checked their concordance with the treatments they were currently receiving, clarifying any reasons for poor concordance. If there were current symptoms of depression, alternative or additional treatments were discussed. These could be pharmacological, psychological or social, with the rationale and evidence for any of these being made clear, both in the background literature given to patients and in their discussion with the nurses. Social factors, which could be contributing to the chronicity of patients' depression, were explored (e.g. social isolation, low physical activity, unemployment, finance, housing) and appropriate advice given or referrals to other agencies made. The importance of patient choice and active participation in this process and in the treatments selected was emphasised.

A joint management plan was then formulated between the nurses and each of their patients and reviewed during subsequent appointments, together with a review of how the patient was feeling and any progress made with previous goals set. We aimed to teach participants how to monitor their own mental state and to have a sense of possible predictors of relapse. Evidence from other studies indicates this can be helpful [15].

### Timing of intervention appointments

Intervention patients were seen for a baseline assessment, after one month and then two months later - i.e. three times over the initial 3 months of the study. The purpose of having the initial appointments more closely spaced was to allow sufficient time for the nurses to get to know the patient well and to formulate a clear management plan together. After this, the reviews for intervention patients took place 3 monthly for the remainder of the 24 month trial period, but could be more frequent if there were any significant clinical concerns about the patients' mood. Such a flexible approach was found helpful in a two-year study with good outcomes, and the arrangements were left to the discretion of the nurse [15]. If patients were keeping well, it was considered appropriate to conduct this review over the telephone, with evidence from other studies showing this to be feasible [23]. Nevertheless, we asked the nurses to have face-to-face contact with participants every few appointments. If nurses were concerned about a patient, they were asked to discuss them with the relevant GP, who might also see the patient if indicated.

### Treatment as usual

During the 24 month study period the participants in the control arm had 'treatment as usual' and continued to see their GP on request, with no restrictions placed on any interventions which the GP might recommend. It was stipulated that the control arm participants should not see the practice research nurse for any mental health intervention, although they might see her for physical health problems.

### Research nurse training sessions

The research team provided 4 full days training for all the participating practice nurses as follows:

• Day 1: covered the procedures required to recruit participants to the trial, checking their eligibility (including conducting the CIDI interview) and conducting the computerised randomisation.

• Day 2: covered the procedures and information required for the intervention appointments, including assessment of a patient's level of depression, details of evidence based pharmacological and psychological treatments for depression, and the importance of considering relevant social factors.

Brief training was given in problem solving techniques to help patients address some of these difficulties.

• Day 3: A further day's training was arranged after 6 months for the nurses to discuss some of their clinical cases, and in particular to focus on ways of working with their more complicated or resistant clients. Brief training was given in the use of simple motivational interviewing techniques to use with patients finding it difficult to make any changes in their lives [24].

All the information from the three training days was written in a manual for the nurses to take away with them, and they were regularly updated with information about potentially useful resources available. The nurses were also encouraged to ensure that they maintained access to up to date information about appropriate local voluntary and other organisations which they could encourage patients to contact where appropriate.

• Day 4: This training session was for the procedures required for the final assessment at 24 months, including conducting a further CIDI interview (see further details below).

### Clinical Supervision sessions

Each nurse was assigned a member of the research team as a 'clinical supervisor' (two of these were general practitioners with a special interest in mental health and one was a clinical psychologist). Nurses had regular telephone contact (generally every 3-4 months) with their supervisors throughout the trial period. The format used was a general review, giving nurses the opportunity to discuss their intervention cases and any concerns they might have, as well as the supervisor making suggestions about possible changes in approach or treatment where appropriate. Nurses could also contact their clinical supervisor in between if they had a particular concern or query about a patient.

### Outcome measures

**The primary outcome measure **is the Beck Depression Inventory (BDI-II). The BDI-II is a reliable and well-validated measure for measurement of the severity of depression and monitoring its clinical outcome, which has been used in many primary care studies [21]. It will also be involved in the assessment of cost-effectiveness as part of the health economic analysis.

### Secondary outcomes (table 1)

**Table 1:** Frequency of measuring outcome measures

	BDI-II	WASAS	CIDI (24 months)	SAPAS	EQ-5D	CSRI	Service Data (24 months)
**Baseline**	√	√	√	√	√	√	√
3 months*	√						
**6 months**	√						
**12 months**	√						
**18 months**	√						
**24 months**	√	√	√		√	√	√

* We had initially planned to collect BDI-II questionnaires 3 monthly, but decided this was too onerous for participants and risked a poor response rate, so this was altered to 6 monthly from the 6 months time-point

(a) Depression-Free Days: These will be assessed in two ways. They will be calculated using BDI-II assessment scores following the method of Lave et al. [25]. This will then be compared with patients' self-completed records of which days they felt they were significantly depressed, to see how a continuous form of data collection compares with a statistical method for assessing persistence of depression. (Our feasibility data indicates that patients will be prepared to keep such on-going records).

(b) Social Functioning: This will be measured using the Work and Social Activity Scale (WASAS). This is a well-established, brief questionnaire, which we will use to assess participants' difficulties with physical and social functioning associated with their depression [26]. It has been validated for use in primary care populations with depression.

(c) Frequency of Depressive Episodes: Will be collected using the CIDI questionnaire [20]. This instrument is frequently used in psychiatric epidemiological studies and has been modified to allow us to collect diagnostic data on trial participants as regards their depression using DSM-IV criteria for the 36 months before recruitment as well as the 24 months of the trial.

(d) Patient personality factors: Participants are asked at baseline to complete the Standardised Assessment of Personality - Abbreviated Scale (SAPAS), a brief measure of personality variables developed for use in primary care [27].

(e) Quality of life: This will be measured by the Euroquol EQ-5 D, which will generate scores for the cost-utility analysis [28].

(f) Resource use and costs: Data on all services used and productivity losses over a retrospective 3-month period will be collected using a modified version of the Client Service Receipt Inventory (CSRI), which has been used in numerous economic evaluations, including in primary care populations [29].

(g) Practice service data: The research nurses will also be asked to count the number of GP attendances and home visits, practice nurse contacts, referrals for psychological therapy and prescriptions for psychotropic medication for all participants for the 24 months before recruitment and the 24 months of the trial.

In order to maintain blindness the final research assessment will be carried out by a different person. This may be another nurse at the practice, a MRC GPRF regional training nurse or a Mental Health Research Network Clinical Studies Officer. All participants will be asked at the outset of the final assessment not to reveal their trial arm allocation. As a check on potential bias, each practitioner assessing outcome will be asked to record which trial arm they think each patient they have assessed was in, to see whether the probability of a correct 'guess' is greater than chance.

### Depression-free Days recorded in Mood Diaries

Following successful piloting in the feasibility study all participants were asked to complete regular mood diaries, indicating for each day whether they had been depressed or not. This was something that had been both feasible and acceptable for the six months of the pilot study, but proved overly time-consuming and onerous for many participants in the full trial, with the response rate being quite poor after the first 3 months.

We therefore reduced the requirement to completion of the diaries for 50% of the project time. An algorithm was constructed, with the 2 years of the trial follow-up divided into eight 3 month portions and participants randomly allocated to receiving the diaries for four of these time periods. Random allocation of diary periods means that data can be imputed for the whole trial period and used as a patient record of their depression free days, as well to validate the scores calculated using the BDI-II scores by the method of Lave et al. [25].

The questionnaires listed were self-completed, apart from the CIDI which was administered by the research nurses in the practices, who also collected the service usage data. In order to ensure good response rates, participants were given a booklet with all the self-completion questionnaires to fill in when they attended the surgery at baseline and at 24 months for their assessments.

BDI-II questionnaires and mood diaries during the intervening two years were posted to all participants with a self-addressed envelope enclosed for their reply. A reminder letter and copy of the questionnaire was sent after two weeks if participants did not respond. If they failed to return the primary outcome measure, the BDI-II, they were telephoned by the practice research nurse to prompt them to return the questionnaire.

### Proposed sample size

We conducted two sample size calculations - for both a 4 and 5 point difference in the primary outcome measure, the BDI-II. In order to detect a 4-point difference on the BDI-II (assuming a pooled standard deviation of 11.0) at 90% power and the 5% level of significance, the required sample size was 318. To meet the objectives of the economic analysis (see details below) data from a previous trial comparing GP care and talking therapies showed a sample size of approximately 200 in each study arm would be required, rising to 534 using an estimated 25% drop-out rate [30].

To take account of practice clustering we used an intra-class correlation (ICC) of 0.02 which, given an estimated 12 to 14 patients recruited per practice, inflated our sample size to 630 from 47 practices (see table 2 below). We considered this to be a suitable ICC given that a primary care trial with the BDI as the outcome for GPs using cognitive behavioural techniques with their patients gave an ICC of 0.13 [31].

**Table 2:** Four Point Difference in BDI

ICC	Design effect*	Total sample size		Number of practices required
		Required	With 25% drop-out	
0	1	400	534	40
0.01	1.09	436	581	44
**0.02**	**1.18**	**472**	**630**	**47**
0.03	1.27	508	678	51
0.04	1.36	544	725	54
0.05	1.45	580	774	58

*Design effect = 1 + (n-1) × ICC, based on clusters with 10 individuals per cluster.

A 4 point difference in BDI score would be a conservative result and is the smallest difference we would expect to achieve. In a previous trial of counselling in primary care the results showed a 5 point difference in outcome which is equivalent to a clinically important treatment effect of 0.5 [31].

We therefore repeated the same calculations for a difference in outcome of 5 points on the BDI, which indicated that we would need 423 participants from as few as 32 practices recruiting 12 to 14 patients each. This would also provide sufficient power for a cost-effectiveness analysis using this outcome.

### Planned analyses

All data will be double entered, using a reputable data entering service, blind as to participant group.

A description of baseline characteristics will be made between those randomised to the control and intervention groups.

All outcome analyses will include individuals in their randomised arm (intention to treat). Initial descriptive statistics will be used to summarise the differences in outcome between the two groups. We will present means and standard deviations for normally distributed, continuous variables and medians, with inter-quartile ranges, for those not normally distributed. Categorical outcomes will be presented as frequencies and percentages within each of the categories.

Observations at each time point on the same individual will not be independent so a repeated measures generalised linear model approach will be used [32]. This will also allow adjustments to be made for the natural clustering inherent in the study design. Continuous outcomes, which are measured at baseline and at the end of follow-up, will be analysed using analysis of covariance (ANCOVA) to adjust for any differences in baseline values [33]. Results will be presented as adjusted differences in means with 95% confidence intervals and associated p-values. Categorical outcomes will be compared using logistic regression and summarised by comparing the group values of the odds ratios for each outcome using 95% confidence intervals and associated p-values. Statistical techniques will be used to assess the impact of potential missing data [34]. Multiple imputation using a predictive model based approach will be used to impute missing values [35]. No interim or sub-group analyses are planned.

The impact of clustering by GP and/or nurse will be investigated using multi-level modelling. This will ensure that estimated standard errors of the treatment effect will be adjusted for the cluster effects.

### Economic Analysis

The economic evaluation will be conducted from two perspectives: (i) focusing on service costs (public expenditure) and (ii) a societal perspective including service costs, productivity losses and informal care inputs. Service costs will be calculated by attaching unit costs to data reported on the CSRI using, wherever possible, national unit costs from publicly-available sources or estimated using an equivalent approach [36,37]. The service use patterns and associated costs for the two groups will be compared at each time point and changes over time will be analysed using standard non-parametric techniques.

Cost-effectiveness will be assessed using the net-benefit statistic, a reformulation of the cost-effectiveness decision rule that does not rely on cost-effectiveness ratios with their associated statistical problems [38]. This will allow the cost-effectiveness analysis to be formulated within a standard regression type framework [39]. Inclusion of the EQ-5 D allows a cost-utility analysis and a cost-effectiveness analysis will link total costs with the primary clinical outcome measure (change in the level of depression using the Beck Depression Inventory-II) and the number of depression-free days (see above). A cost-consequence analysis will explore the relationship between cost, characteristics, needs and outcome measures [40]. All analyses will take into account cluster effects in practices and sensitivity analyses will check assumptions made in the cost calculations and analyses.

### Interim results - Participant recruitment

**Figure 2:**
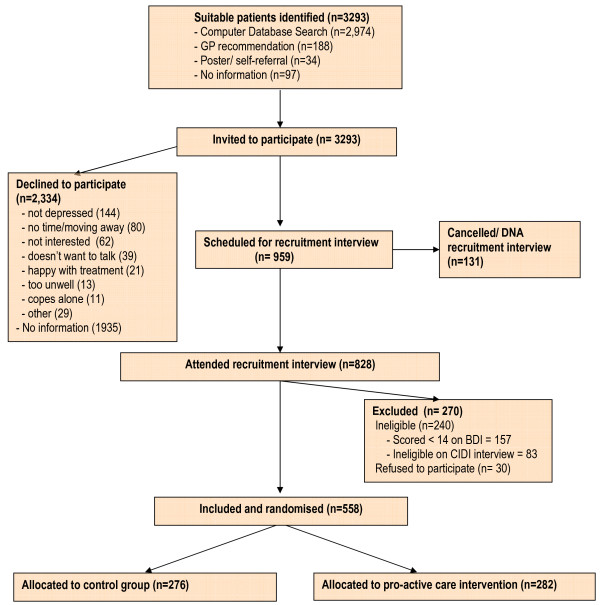
**Consort Diagram: Recruitment and treatment group allocation**.

3,293 people in the 42 participating practices were identified as potentially suitable to take part in the trial using the methods described above and were sent letters from their practices giving them information about the study and inviting them to come and discuss this in more detail with the practice research nurses. Most of these potential participants (2,974) were identified via database searches, and only small numbers via GP or nurse identification (188) or self-referral in response to practice posters (34). See Figure 2, the Consort Diagram.

Of the 3,293 people initially approached, 959 (29%) expressed an interest in attending for the interview and 828 (25%) actually attended. Following the recruitment interview and assessment 558 people were found eligible and agreed to take part in the trial. Using the computerised randomisation system 276 participants were allocated to the Control Group and 282 to the Intervention Group. 240 people were found to be ineligible at the recruitment interview stage, 157 because they did not score at least 14 on the BDI-II and 83 because they were ineligible on the CIDI.

### Use of Vouchers

Because the response rate for the primary outcome measure the BDI-II went down from 72% to 66% between the 3 months and 6 months follow-up points, there was a concern that this would persist or get worse at later time points and impair the validity of the study results. We therefore decided to reduce the frequency of the BDI-II questionnaires to 6 monthly (see frequency table; table 1 above) and to incentivise completion by sending participants who had returned questionnaires a £ 10 High Street voucher which can be used at many stores nationally. Ethical approval was granted for sending of the vouchers, which represented a reimbursement to the participants for time spent completing and returning the questionnaires.

At 12 months the BDI-II response rate was 67%, which was still not optimal. We therefore changed our approach at the 18 month time-point and sent the vouchers at the same time as the BDI-II questionnaires, as a recent Cochrane review indicated that this may further improve the response rate [41].

### Ethical Approval

This trial received ethical approval from the Royal Free Hospital & Medical School Research Ethics Committee on the 21^st ^February 2007 - REC reference number 07/Q0501/15. Amendments were approved in July 2007, November 2008 and May 2009.

## Discussion

This RCT recruited 558 participants within the UK over a 9-month period. By far the most effective recruitment method was to use structured searches of general practice databases for potential participants, who were then approached through a letter, signed by a GP and nurse at the practice, which told them about the study and asked if they were interested in taking part. Much smaller numbers of participants were recruited through GP or nurse recommendations or the leaflets or posters in the surgery encouraging self-referral.

The numbers recruited should allow us to demonstrate a clinically significant effect if the intervention is to be helpful in this population with its significant psychological and functional morbidity. We will review the effect of sending participants vouchers at the 12, 18 and 24 month time-points to see whether this has had the desired effect of improving the questionnaire return rate.

## Abbreviations

GP: General Practitioner; MRC GPRF: Medical Research Council General Practice Research Framework; CIDI: Composite International Diagnostic Interview; BDI-II: Beck Depression Inventory II; DSM IV: Diagnostic and Statistical Manual of Mental Disorders, 4^th ^edition; WASAS: Work and Social Activity Scale; SAPAS: Standardised Assessment of Personality - Abbreviated Scale; CSRI: Client Service Receipt Inventory

## Competing interests

The authors declare that they have no competing interests.

## Authors' contributions

MB, MG and MK were involved in the original design and submission of the protocol for funding and JB reviewed the protocol from a health economics perspective. EM was involved, together with MB, in setting up and running the trial from its beginning. All authors have been involved in giving detailed comments on this paper.

## Pre-publication history

The pre-publication history for this paper can be accessed here:

http://www.biomedcentral.com/1471-244X/10/61/prepub
